# Circulating Visfatin in Hypothyroidism Is Associated with Free Thyroid Hormones and Antithyroperoxidase Antibodies

**DOI:** 10.1155/2016/7402469

**Published:** 2016-01-17

**Authors:** Nadia Sawicka-Gutaj, Ariadna Zybek-Kocik, Aleksandra Klimowicz, Michał Kloska, Dorota Mańkowska-Wierzbicka, Jerzy Sowiński, Marek Ruchała

**Affiliations:** ^1^Department of Endocrinology, Metabolism and Internal Medicine, Poznan University of Medical Sciences, Przybyszewski Street 49, 60-355 Poznań, Poland; ^2^Department of Gastroenterology, Human Nutrition and Internal Diseases, Poznan University of Medical Sciences, Przybyszewski Street 49, 60-355 Poznań, Poland

## Abstract

We hypothesized that regulation of visfatin in hypothyroidism might be altered by coexisting chronic autoimmune thyroiditis. This is a prospective case-control study of 118 subjects. The autoimmune study group (AIT) consisted of 39 patients newly diagnosed with hypothyroidism in a course of chronic autoimmune thyroiditis. The nonautoimmune study group (TT) consisted of 40 patients thyroidectomized due to the differentiated thyroid cancer staged pT1. The control group comprised 39 healthy volunteers adjusted for age, sex, and BMI with normal thyroid function and negative thyroid antibodies. Exclusion criteria consisted of other autoimmune diseases, active neoplastic disease, diabetes mellitus, and infection, which were reported to alter visfatin level. Fasting blood samples were taken for visfatin, TSH, free thyroxine (FT4), free triiodothyronine (FT3), antithyroperoxidase antibodies (TPOAb), antithyroglobulin antibodies (TgAb), glucose, and insulin levels. The highest visfatin serum concentration was in AIT group, and healthy controls had visfatin level higher than TT (*p* = 0.0001). Simple linear regression analysis revealed that visfatin serum concentration was significantly associated with autoimmunity (*β* = 0.1014; *p* = 0.003), FT4 (*β* = 0.05412; *p* = 0.048), FT3 (*β* = 0.05242; *p* = 0.038), and TPOAb (*β* = 0.0002; *p* = 0.0025), and the relationships were further confirmed in the multivariate regression analysis.

## 1. Introduction

Visfatin, also known as nicotinamide phosphoribosyltransferase (NAMPT) as well as pre-B-cell colony-enhancing factor, is a multifaceted protein with suggested enzymatic, immunological, and metabolic properties. Visfatin has been analyzed in hypo- and hyperthyroidism in* in vitro* and* in vivo* studies, but results are inconclusive [1]. In addition, NAMPT level was found to be elevated in many autoimmune diseases, that is, rheumatoid arthritis, systemic lupus erythematosus, inflammatory bowel diseases, and psoriasis [2–5]. Visfatin also positively correlates with activity and severity of rheumatoid arthritis and psoriasis [2, 5]. We have recently found an overexpression of NAMPT in leukocytes of patients with Graves' ophthalmopathy with corresponding increased serum concentration (accepted manuscript). Our findings suggest that visfatin might be involved in autoimmune processes in thyroid diseases.

In our opinion, the controversial findings of visfatin in thyroid hormone deficiency may arise from the heterogeneity of thyroid dysfunction. We hypothesized that regulation of visfatin in hypothyroidism might be altered by coexisting chronic autoimmune thyroiditis, since high visfatin levels were observed in other autoimmune diseases. To answer the question about the influence of coexisting chronic autoimmune inflammation on visfatin level, we analyzed its serum concentration among hypothyroid patients with chronic autoimmune thyroiditis and in patients after thyroidectomy, who were negative for thyroid antibodies.

## 2. Patients and Methods

### 2.1. Study Design and Patient Recruitment

This is a prospective case-control study of 118 subjects. The autoimmune study group (AIT) consisted of 39 patients newly diagnosed with hypothyroidism in a course of chronic autoimmune thyroiditis. The nonautoimmune study group (TT) consisted of 40 patients thyroidectomized due to the differentiated thyroid cancer staged pT1. TT patients were evaluated five years after radioiodine remnant ablation and were negative for thyroglobulin and radioiodine uptake in a whole body scintigraphy (WBS). They achieved endogenous TSH stimulation and became hypothyroid after L-T4 withdrawal for at least 4 weeks. The control group comprised 39 healthy volunteers adjusted for age, sex, and BMI with normal thyroid function and negative thyroid antibodies. Exclusion criteria consisted of other autoimmune diseases, active neoplastic disease, diabetes mellitus, and infection, which were reported to alter visfatin level. Every patient underwent physical examination and thyroid/neck ultrasound examination.

### 2.2. Laboratory Analysis

Fasting blood samples were taken for visfatin, TSH, free thyroxine (FT4), free triiodothyronine (FT3), antithyroperoxidase antibodies (TPOAb), antithyroglobulin antibodies (TgAb), glucose, and insulin levels. In TT group also thyroglobulin (Tg) level was measured.

Visfatin serum concentration was measured with ELISA Assay Kit from Phoenix Pharmaceuticals. TSH, FT4, and FT3 were measured using the electrochemiluminescence technique in Cobas E 601 (norm ranges: TSH 0.27–4.2 mU/L; FT4 11.5–21.0 pmol/L; FT3 3.93–7.70 pmol/L). TPOAb and TgAb were measured by radioimmunoassay (norm range: <34 IU/mL and <115 IU/mL, resp.). Glucose level was assessed with the use of Hitachi Cobas e601 chemiluminescent analyzer (Roche Diagnostics) and insulin concentration was assessed using ELISA kit from Phoenix Pharmaceuticals. The estimate of insulin resistance by homeostasis model assessment (HOMA-IR) was calculated.

The study was approved by the local ethics committee, and informed consent was signed by every subject.

### 2.3. Statistical Analysis

Statistical analysis was performed with MedCalc version 12.1.3.0 (MedCalc Software, Mariakerke, Belgium). Normality was analyzed by D'Agostino-Pearson test. Variables with normal distribution were compared between three groups with one-way analysis of variance. If data did not follow normal distribution, comparison of the analyzed parameters between three groups was performed with the Kruskal-Wallis test. Simple regression analysis was used to test for the relationships between them. Before inclusion to this statistical analysis, nonnormally distributed parameters were logarithmically transformed. Furthermore, stepwise multiple regression analysis was employed to investigate the influence of various parameters on visfatin serum concentration [age, BMI, FT3, autoimmunity (yes/no), HOMA-IR]. Variables were entered into the model if their associated *p*-values were less than 0.05 and then sequentially removed if their associated *p*-values became greater than 0.2. Tests were considered to be statistically significant if *p*-value was lower than 0.05.

## 3. Results

Clinical and laboratory data of the study groups and the control group are shown in Table 1.

**Figure 1:**
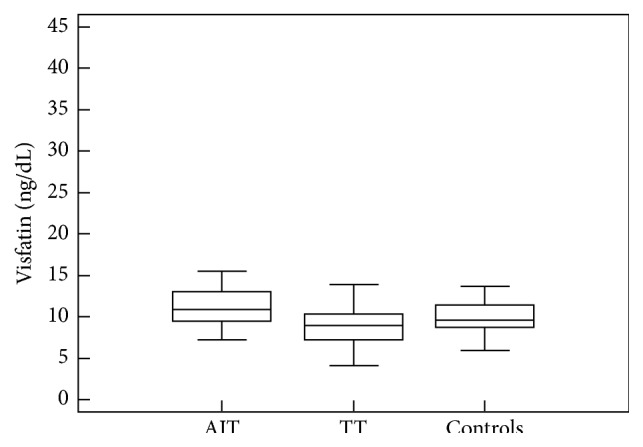
Comparison of visfatin serum concentration in hypothyroid patients with chronic autoimmune thyroiditis (AIT), in hypothyroid patients after total thyroidectomy (TT), and in healthy controls (controls). Central box represents the values from the lower to upper quartile (25th to 75th percentile). The middle line represents the median. The thin vertical lines extending up or down from the boxes to horizontal lines (so-called whiskers) extend to a multiple of 1.5 × the distance of the upper and lower quartile, respectively.

The highest visfatin serum concentration was in AIT group, and healthy controls had visfatin level higher than TT (*p* = 0.0001) (Figure 1). Three groups did not differ in age, sex, BMI, fasting glucose, and insulin levels, HOMA-IR. They had statistically different TSH, FT4, FT3, and TgAb levels (Table 1). AIT group had higher TPOAb level. TT and controls were negative for TPOAb and TgAb.

**Figure 2:**
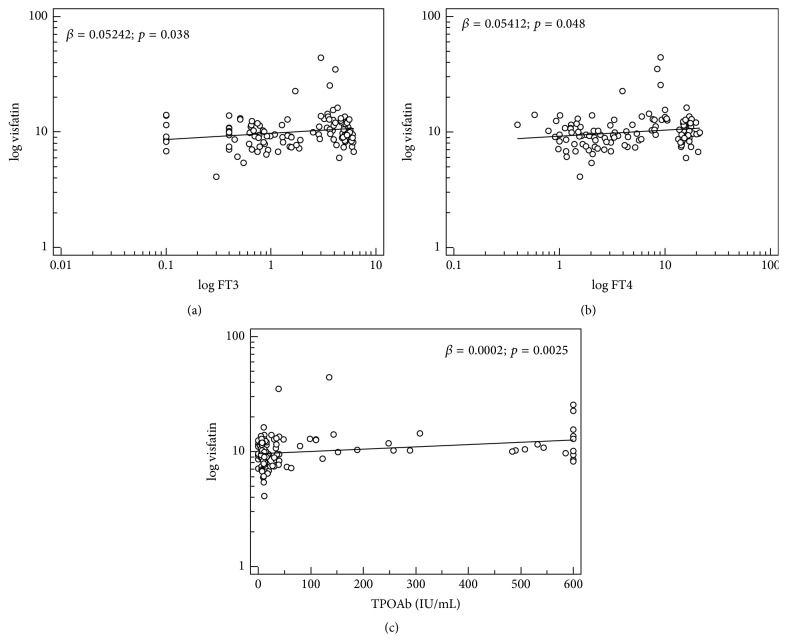
Association between visfatin serum level and FT3 (a), FT4 (b), and TPOAb (c). Data were log-transformed to achieve normal distribution.

Simple linear regression analysis revealed that visfatin serum concentration was significantly associated with autoimmunity (*β* = 0.1014; *p* = 0.003), FT4 (*β* = 0.05412; *p* = 0.048), FT3 (*β* = 0.05242; *p* = 0.038), and TPOAb (*β* = 0.0002; *p* = 0.0025) (Figure 2). There was no association between visfatin and age, sex, BMI, TSH, TgAb, fasting insulin and glucose levels, and HOMA-IR (Table 2).

In the stepwise multiple regression analysis we confirmed the association between serum visfatin level and autoimmunity (coefficient = 3.8461; *p* = 0.0001), and FT3 (coefficient = 0.4198; *p* = 0.0441), whereas age, BMI, and HOMA-IR did not contribute significantly. In separate stepwise multiple regression analysis we confirmed the association of serum visfatin concentration with autoimmunity (coefficient = 4.1105; *p* = 0.0001) and FT4 (coefficient = 0.1397; *p* = 0.038), whereas age, BMI, and HOMA-IR did not enter the model. Similarly, association of visfatin with TPOAb (coefficient = 0.0057; *p* = 0.0163) was observed with adjustment for age, BMI, FT3, and HOMA-IR in multivariate regression analysis.

## 4. Discussion

To date, visfatin serum concentration in hypothyroidism has been analyzed in a few studies [1]. Caixàs et al. reported elevated level of this adipocytokine in hypothyroidism with further increase after restoration of thyroid function [6]. Ozkaya et al. observed that visfatin level decreased after recovery [7]. In those articles the etiology of hypothyroidism varied from chronic autoimmune thyroiditis, postpartum thyroiditis to thyroid function insufficiency after radioiodine treatment or after thyroidectomy. Hence, to date autoimmune status of studied patients has not been taken into consideration. We hypothesized that these controversial findings might result from heterogeneity of study groups.

To answer the question, whether coexisting autoimmune inflammation influences visfatin level in hypothyroid patients, we analyzed its serum concentration in chronic autoimmune thyroiditis and thyroidectomized patients negative for thyroid antibodies. Since we have previously proved that visfatin mRNA expression is increased in thyroid malignancies and is correlated with tumor stage, we recruited only those patients who did not have any features of active neoplastic disease [8]. We also recruited the control group adjusted for age, sex, and BMI. To the best of our knowledge, this is the first study addressing the changes in the release of visfatin in thyroid autoimmunity. We came to interesting results indicating that visfatin serum concentration in hypothyroid patients is associated with both autoimmunity and free thyroid hormones level (FT4, FT3). We confirmed our findings in adjusted models.

Visfatin has been recognized as a cytokine with a broad range of immune and inflammatory activities, including induction of inflammatory cytokines, and regulation of macrophage and lymphocyte proliferation [9]. Visfatin stimulates the production of proinflammatory cytokines (IL-6, TNF-*α*, and IL-1*β*) and potentially acts as a chemotactic factor for monocytes. Furthermore, its expression is upregulated by IL-6, TNF-*α*, and IL-1*β* [10–12]. Enhanced mRNA expression of visfatin was observed in inflamed mucosa of patients with inflammatory bowel disease (IBD) [4]. Further analysis identified that antigen presenting cells (i.e., macrophages, dendritic cells) might be a main source of this protein in IBD. Visfatin has also potency for activation of T cells by upregulation of costimulatory molecules (CD40, CD54, and CD80) on monocytes. We observed the positive association between visfatin and TPOAb, and the latter is considered the best serological marker of chronic autoimmune thyroiditis. Furthermore, TPOAb contribute to thyroid destruction through antibody- and complement-dependent cell-cytotoxicity [13, 14]. The first mechanism is associated with mononuclear cell infiltration of thyroid stroma. In addition, Th1-derived cytokines (Il-2, TNF-*α*, and INF) were found to be elevated in patients with chronic autoimmune thyroiditis [15]. Also, Il-1*β* and TNF-*α* have been recently reported to discriminate chronic autoimmune hypothyroid children from healthy controls [16]. Altogether, association of visfatin with TPOAb observed in our study further supports our hypothesis that visfatin might be involved in the pathogenesis of chronic autoimmune thyroiditis.

We also reported that visfatin serum concentration depends on FT3 and FT4. Our findings about the association of visfatin with FT3 levels are in accordance with the results of* in vitro* and* in vivo* studies. However, controversial results whether T3 stimulates or downregulates the production of visfatin were found. Ozkaya et al. showed the significant negative correlation between visfatin and FT3 [7]. In contrast, Caixàs et al. did not find any relationship between visfatin and free thyroid hormones [6].* In vitro* experiment showed the nonlinear regulation of visfatin mRNA expression in the 3T3-L1 cell culture model affected by T3 [17]. Since our study groups significantly differed with free thyroid hormones levels, we were able to analyze visfatin concentration in a broad spectrum of FT3 and FT4. Therefore, we might suggest that pattern of visfatin changes varies in different FT3 concentration.

According to our observations, as well as other authors, visfatin did not reflect insulin resistance assessed by HOMA-IR [18, 19]. Although, there are studies confirming this relationship [20, 21]. The metabolic role of visfatin remains unclear.

Our study might then prove that visfatin in hypothyroidism depends on thyroid hormones level and coexisting autoimmunity. We may assume that these two factors should be taken into consideration to assess visfatin level in patients with thyroid dysfunction. In addition, the possible involvement of visfatin in pathogenesis of chronic autoimmune thyroiditis needs further research.

## Limitation of the Study

The main limitation of our study is its cross-sectional design that does not enable us to reveal the causal pathways of relationship between visfatin and autoimmune thyroiditis. However, we used very strict criteria of exclusion to limit the possible influence of other known factors such as diabetes mellitus, other autoimmune processes, infection, and active neoplastic disease. Our nonautoimmune group with hypothyroidism had been thyroidectomized at least 5 years earlier and did not have any clinical nor laboratory features of active thyroid cancer.

## Figures and Tables

**Table 1:** Comparison of clinical and laboratory characteristics between the study groups and controls.

	Autoimmune hypothyroidism (*n* = 39)	Nonautoimmune hypothyroidism (*n* = 40)	Controls (*n* = 39)	*p*
Sex (F—females; M—males)	F 34 M 5	F 37 M 3	F 34 M 5	0.5420
Age [yr] median (IQR)	46 (34.5–54)	41.5 (29.5–48)	43 (36.3–54)	0.1837
BMI [kg/m^2^] median (IQR)	23.4 (21.65–25.4)	25.4 (21.55–27.15)	23.3 (20.73–26.13)	0.2704
Glucose [mg/dL] mean (SD)	93 (9.7)	90.8 (9.6)	90.0 (8.0)	0.319
Insulin [*μ*U/mL] mean (SD)	7.7 (3.4)	8.14 (3.12)	8.99 (3.2)	0.202
HOMA-IR mean (SD)	1.77 (0.77)	1.82 (0.72)	2.02 (0.82)	0.316
TSH [*μ*U/mL] median (IQR)	57.3 (35.4–100)	94.5 (62.13–100)	1.8 (1.38–2.57)	**<0.0001**
FT4 [pmol/L] median (IQR)	5.4 (2.16–8.16)	1.84 (1.34–2.67)	15.96 (14.8–17.39)	**<0.0001**
FT3 [pmol/L] mean (SD)	2.28 (1.4)	0.7 (0.37)	5.15 (0.57)	**<0.001**
TPOAb [IU/mL] median (IQR)	189 (56.25–574.5)	11 (7–16)^a^	9 (6–12.75)^a^	**<0.001**
TgAb [IU/mL] median (IQR)	267 (79–504)	17 (13–21)	10 (10–14)	**<0.0001**
Tg [ng/mL] mean (SD)	—	0.4 (0.34)	—	—
Visfatin [ng/mL] median (IQR)	10.85 (9.52–13.03)	8.97 (7.25–10.3)	9.54 (8.68–11.4)	**0.0001**

^a^Values followed by the same letter do not differ significantly.

TPOAb antithyroperoxidase antibodies; TgAb antithyroglobulin antibodies; Tg thyroglobulin.

**Table 2:** Simple linear regression analysis using visfatin serum concentration as dependent variable.

Variable	Visfatin serum concentration (log)	
	*β*	*p*
Age	−0.001	0.3325
Sex	−0.0229	0.564
BMI (log)	−0.3624	0.059
TSH (log)	−0.0046	0.784
FT4 (log)	**0.05412**	**0.048**
FT3 (log)	**0.05242**	**0.038**
TPOAb	**0.0002**	**0.0025**
TgAb	0.00001	0.515
Fasting glucose	0.0009	0.489
Fasting insulin	−0.0026	0.518
HOMA-IR (log)	−0.0006	0.720
Autoimmunity (yes/no)	**0.1014**	**0.0003**

TPOAb antithyroperoxidase antibodies; TgAb antithyroglobulin antibodies.
